# Clinical and molecular characterization of 112 single-center patients with Neurofibromatosis type 1

**DOI:** 10.1186/s13052-018-0483-z

**Published:** 2018-04-04

**Authors:** Giovanni Corsello, Vincenzo Antona, Gregorio Serra, Federico Zara, Clara Giambrone, Luca Lagalla, Maria Piccione, Ettore Piro

**Affiliations:** 1 Department of Sciences for Health Promotion and Mother and Child Care “G. D’Alessandro”, University of Palermo, Via A. Giordano 3, 90127 Palermo, Italy; 2 Laboratory of Neurogenetics and Neuroscience, Institute G. Gaslini, Via Gerolamo Gaslini 5, 16100 Genoa, Italy

**Keywords:** *NF1* gene, Genotype-phenotype correlation, New mutation, *NF1* microdeletion syndrome

## Abstract

**Background:**

The aim of this retrospective study was to define clinical and molecular characteristics of a large sample of neurofibromatosis type 1 (NF1) patients, as well as to evaluate mutational spectrum and genotype-phenotype correlation. NF1 is a relatively common neurogenetic disorder (1:2500–1:3000 individuals). It is caused by mutations of the *NF1* gene on chromosome 17ql1.2, with autosomal dominant pattern of inheritance and wide phenotypical variability. Café-au-lait spots (CALs), cutaneous and/or subcutaneous neurofibromas (CNFs/SCNFs), skinfold freckling, skeletal abnormalities, Lisch nodules of the iris and increased risk of learning and intellectual disabilities, as well as tumors of the nervous system and other organs are its main clinical features.

**Methods:**

The preliminary group collected 168 subjects with clinical suspicion of NF1. They were evaluated following the National Institutes of Health (NIH) criteria for NF1, revised by Gutmann et al. 1997, integrated for 67 of them by molecular testing. According to these references, 112 of 168 patients were diagnosed as NF1. The sample was characterized by an equal sex ratio (57 males, 55 females) and age distribution ranging from 10 days to 60 years of age (mean age, 13 years).

**Results:**

A wide spectrum of clinical features has been observed in our patients. Mutational analysis resulted positive in 51 cases (76%). Twenty-four mutations detected in our cohort have not been reported to date.

**Conclusions:**

This study may contribute to a better definition of genotypic and phenotypic features of NF1 patients, with respect to further insights into the clinical characterization of the disease. In addition, an amplification of the spectrum of mutations in the *NF1* gene has been documented.

## Background

The term RASopathy, firstly used in 2009, defines a group of syndromes caused by germline mutations in genes that encode components or regulators of the Ras/mitogen-activated protein kinase (MAPK) pathway [1]. These syndromes include: neurofibromatosis type 1 (NF1), Noonan syndrome (NS), NS with multiple lentigines, Legius syndrome, Costello syndrome, cardio-facio-cutaneous syndrome (CFC) and capillary malformation arteriovenous malformation (CM-AVM) [2, 3]. NF1 is a relatively common neurogenetic disorder [4]. Its incidence is about 1:2500–1:3000 individuals worldwide, with no differences in sex or ethnic origin [5]. It is caused by mutations of the *NF1* gene on chromosome 17ql1.2, which encodes neurofibromin. NF1 shows an autosomal dominant pattern of inheritance and wide phenotypical variability. Café-au-lait spots (CALs), cutaneous and/or subcutaneous neurofibromas (CNFs/SCNFs), skinfold freckling, skeletal abnormalities and Lisch nodules of the iris are its main clinical features [6]. NF1 patients have an increased risk of learning and intellectual disabilities as well as tumors of the nervous system and other organs [7]. The neoplastic risk is related to functional loss of Ras GTPase-activating protein determining sustained activation of the Ras/Raf/ERK pathway [8]. Since unpredictability of the clinical evolution of disease and possible complications, a reliable genetic counselling is hampered and most of the genotype-phenotype correlation attempts have been unsuccessful [8]. However, with the advent of recent genetic testing, pathogenic correlations emerge at least for those subjects carrying genomic deletions of the entire *NF1* gene, showing more severe phenotype [4]. The aim of the present retrospective study was to evaluate both mutational and phenotypical spectra in a large cohort (*N* = 112) of clinically well-characterized NF1 patients, also in view of a genotype-phenotype correlation.

## Methods

### Patients

Clinical and genetic data were obtained from a cohort of NF1 patients referred to the Mother and Child Department of the University of Palermo, observed between January 2012 and December 2017. The study was approved by the local ethical committee, with participants giving their informed consent. Genomic DNA was extracted from the patient’s peripheral blood lymphocytes and analyzed. The preliminary patients group collected 168 subjects, carried to the geneticist’s evaluation with the clinical suspicion of NF1. The age distribution at diagnosis is shown in Table 1. Most of the cases were ranging between 5 and 18 years (70%). Clinical data were obtained from medical records over the past six years, and the clinical/instrumental findings from each patient were analyzed in a standardized way. The study includes two individuals bearing a large genomic deletion on chromosome 17q11.2, involving different genes including *NF1*.

**Table 1:** Age distribution at diagnosis

Age distribution at diagnosis				
0–1 year	2–4 years	5–18 years	19–30 years	31–60 years
6 (5%)	11 (10%)	78 (70%)	9 (8%)	8 (7%)

### Patient demographics and clinical characteristics

All patients were clinically evaluated following the National Institutes of Health (NIH) criteria for NF1 [5, 9], revised by Gutmann et al. 1997 [10], integrated for 67 of them by molecular testing. According to these references, 112 of 168 patients were diagnosed as NF1. Fifty-six subjects did not meet the strict NIH clinical diagnostic criteria for NF1, and therefore they were excluded from the statistical analyses. Most of the patients were unrelated (except for 7 couples of siblings). Gender distribution (57 males, 55 females) was equal. Ethnic origin was predominantly Caucasian (only 2 patients of black ethnicity, 1.8%). Age distribution ranged from 10 days to 60 years of age (mean age, 13 years). Follow-up evaluations were individually planned, depending on age, clinical severity and involvement of organs and systems. A multidisciplinary approach was applied for both diagnosis and treatment of patients. Data on auxological parameters, dysmorphic features, skin abnormalities, symmetry and shape of thorax and limbs were obtained and analyzed.

### Mutational analysis

DNA was isolated from the peripheral blood leukocytes, and short-term phytohemagglutinin-stimulated lymphocyte culture was used to extract total RNA. Using genomic DNA samples, all coding exons and intron-exon boundaries of the *NF1* gene were amplified by polymerase chain reaction (PCR), and PCR products were purified and directly sequenced. For identifying deep intronic splice mutations, reverse transcription PCR using extracted RNA samples was performed, and the synthesized complementary DNA was analyzed to confirm the skipping of an adjacent exon to the mutation. When no pathogenic mutations were detected by the assays above, the samples were analyzed using multiplex ligation-dependent probe amplification (MLPA) or fluorescence in situ hybridization (FISH), for detecting the entire gene deletion and multiple exon deletions or duplications. Array comparative genomic hybridization (a-CGH), was reserved for the only 2 cases of *NF1* gene microdeletion syndrome.

## Results

Family history was positive for NF1 in 61.6% of patients (n. 69). 43 patients were sporadic (38.4%). At physical examination, head circumference resulted over 2 standard deviations (SD) in 5.3% of individuals (n. 6). CALs (≥6) were present in 92% of subjects (n. 103, 51 males and 52 females). The prevalence in the various age groups was: 0–1 year 100%, 2–4 years 81.8%, 5–18 years 94.8%, 19–30 years 100% and 31–60 years 62.5%. Freckling was present in 20.5% of subjects (n. 23, 10 males and 13 females). The prevalence in the various age groups was: 0–1 year 33.3%, 2–4 years 27.3%, 5–18 years 16.6%, 19–30 years 44.4% and 31–60 years 12.5%. Neurofibromas were present in 26.7% of patients. Specifically, 19.6% of cases showed CNFs (n. 22, 12 males and 10 females) and 7.1% SCNFs (n. 8, 4 males and 4 females). Plexiform neurofibromas were observed in 3.5% of subjects (n. 4, 3 males and 1 female). Considering the neurofibromas all together, the prevalence in the various age groups was: 0–1 year 50%, 2–4 years 27.2%, 5–18 years 16.6%, 19–30 years 77.7% and 31–60 years 100%.

All patients performed eye examination. Lisch nodules of the iris were detected in 10% of cases (n. 11, 5 males and 6 females). The prevalence in the various age groups was; 0–1 year 0%, 2–4 years 9, 5–18 years 9, 19–30 years 11% and 31–60 years 25%).

**Fig. 1:**
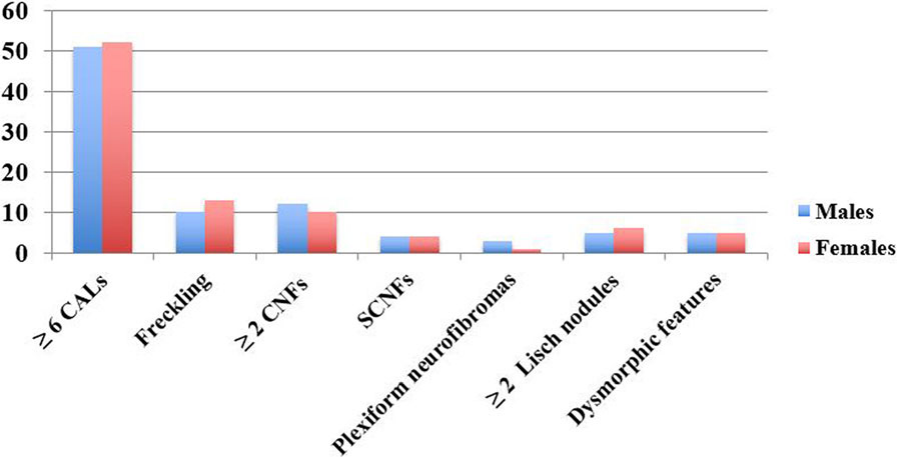
Main clinical features and sex distribution

Dysmorphic features (long face, bitemporal narrowing, wide and protruding ears, hypotelorism, epicanthal folds, prominent glabella, long philtrum, thick lips, micrognathia, dysodontiasis, ogival palate) were observed in 9% of patients (n. 10), all of them bearing wide genomic deletions involving *NF1*. Main clinical features for age groups are reported in Table 2 and Fig. 1.

**Table 2:** Main clinical features distribution and prevalence for age groups

Age groups in years	N^o^	≥6CALs		Ferecling		≥2 CNFs		SCNFs		Plexiform neurofibromas		≥2Lisch nodules		Dysmorphic features	
		N^o^	%	N^o^	%	N^o^	%	N^o^	%	N^o^	%	N^o^	%	N^o^	%
0–1	6	6	100	2	33.3	1	16.6	1	16.6	0	–	0	–	1	16.6
2–4	11	9	81.8	3	27.3	2	18.1	0	–	1	9	1	9	5	45.4
5–18	78	74	94.8	13	16.6	8	10.2	4	5	1	1.3	7	9	4	5.2
19–30	9	9	100	4	44.4	6	66.6	2	22	0	–	1	11	0	–
31–60	8	5	62.5	1	12.5	5	62.5	1	12.5	2	25	2	25	0	–
Total	112	103	92	23	20.5	22	19.6	8	7.1	4	3.5	11	10	10	9

In our sample main clinical features showed an equal sex ratio (Fig. 1)

All patients up to 10 years of age performed developmental and cognitive evaluation. In patients up to 4 years, psychomotor delay or preschool learning difficulties of variable degree, were present in 53% (n. 9/17, 7 males/2 females), this male gender prevalence has statistical significance (*p* = 0.0117). In patients from 5 to 10 years of age, learning or cognitive disabilities leading to school difficulties, were detected in 38% of individuals (n. 13/34, 7 males/6 females).

**Fig. 2:**
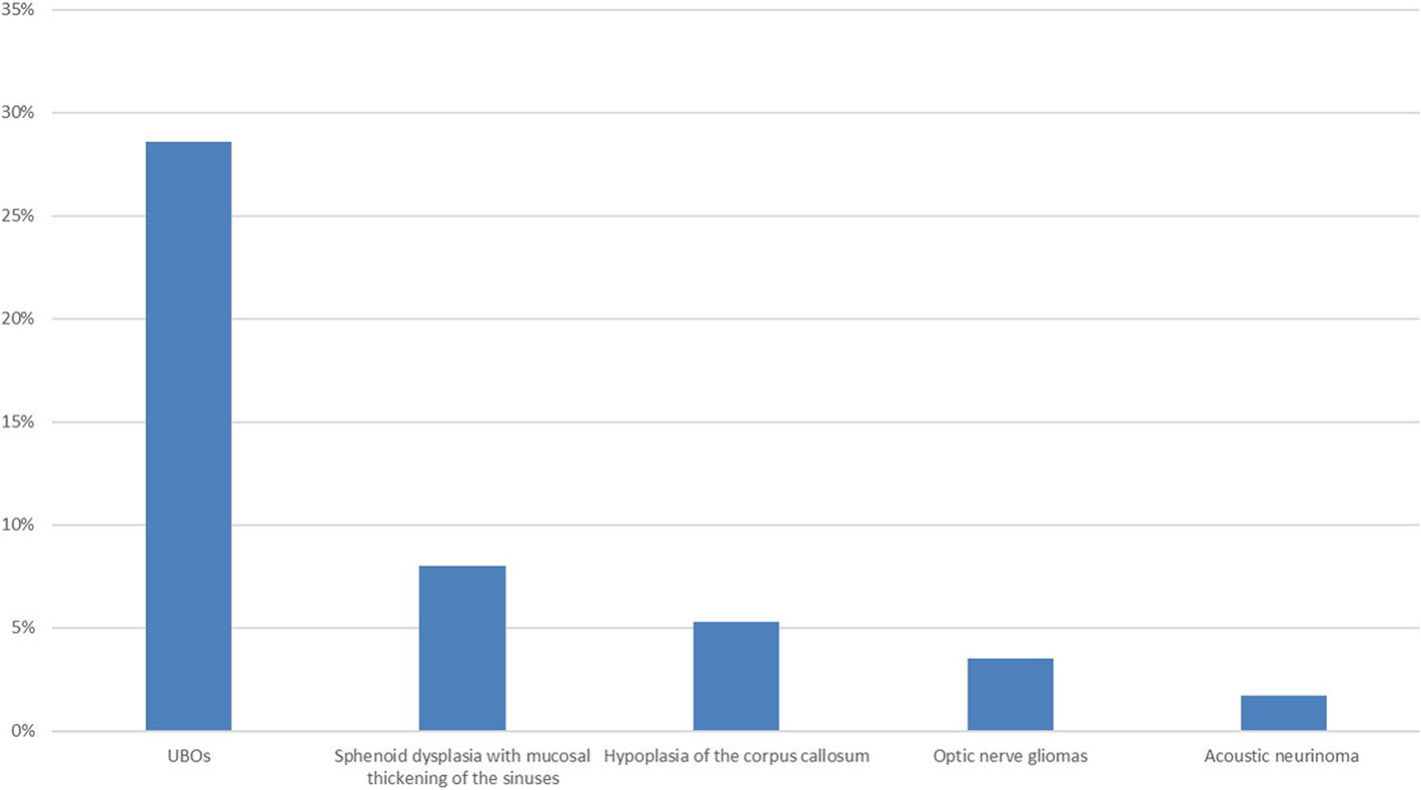
Brain MR abnormalities

All patients performed brain magnetic resonance (MR). Brain anomalies were identified in 47.3% of patients (n. 53). The more frequent radiologic findings were unidentified bright objects (UBOs) (28.6%, n. 32), followed by sphenoid dysplasia with mucosal thickening of the sinuses (8%, n. 9), hypoplasia of the corpus callosum (5.3%, n. 6), optic nerve gliomas (3.5%, n. 4) and acoustic neurinomas (1.7%, n. 2) (Fig. 2).

All patients performed cardiological evaluation and echocardiography. Heart defects were found in 6.2% of cases: mitral valve insufficiency in 1.7% of subjects, interatrial defect/patent *foramen ovale* in 4.4% of patients over 1 year.

All patients performed renal ultrasound. Renal abnormalities were found in 5.3% of individuals: hydronephrosis was present in 2.6%, agenesis/hypoplasia of the kidney in a further 2.6%.

**Fig. 3:**
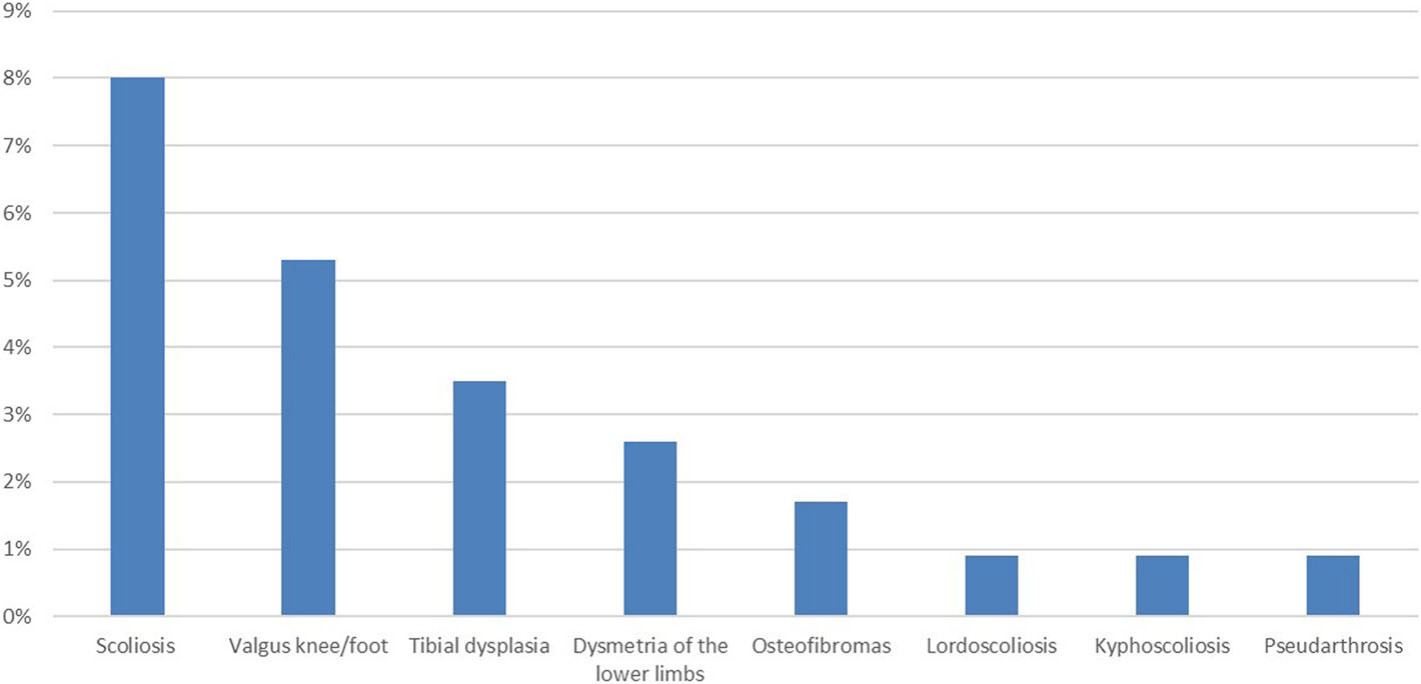
Bone lesions and relative complications

Bone lesions and relative complications were noticed in 24.1% (n. 27) of patients, and listed as follows: scoliosis (8%, n. 9), valgus knee/ft (5.3%, n. 6), tibial dysplasia (3.5%, n. 4), dysmetria of the lower limbs (2.6%, n. 3), osteofibromas (1.7%, n. 2), lordoscoliosis (0.9%, n. 1), kyphoscoliosis (0.9%, n. 1), pseudarthrosis (0.9%, n. 1) (Fig. 3).

Further additional signs and symptoms were: short stature (6.2%, n. 7), headache (4.4%, n. 5), sinusitis (3.5%, n. 4), seizures (3.5%, n. 4), hydrocephalus (2.6%, n. 3), *pectus excavatum* (1.7%, n. 2), angiomas (1.7%, n. 2) and juvenile xantogranulomas (0.9%, n. 1).

**Fig. 4:**
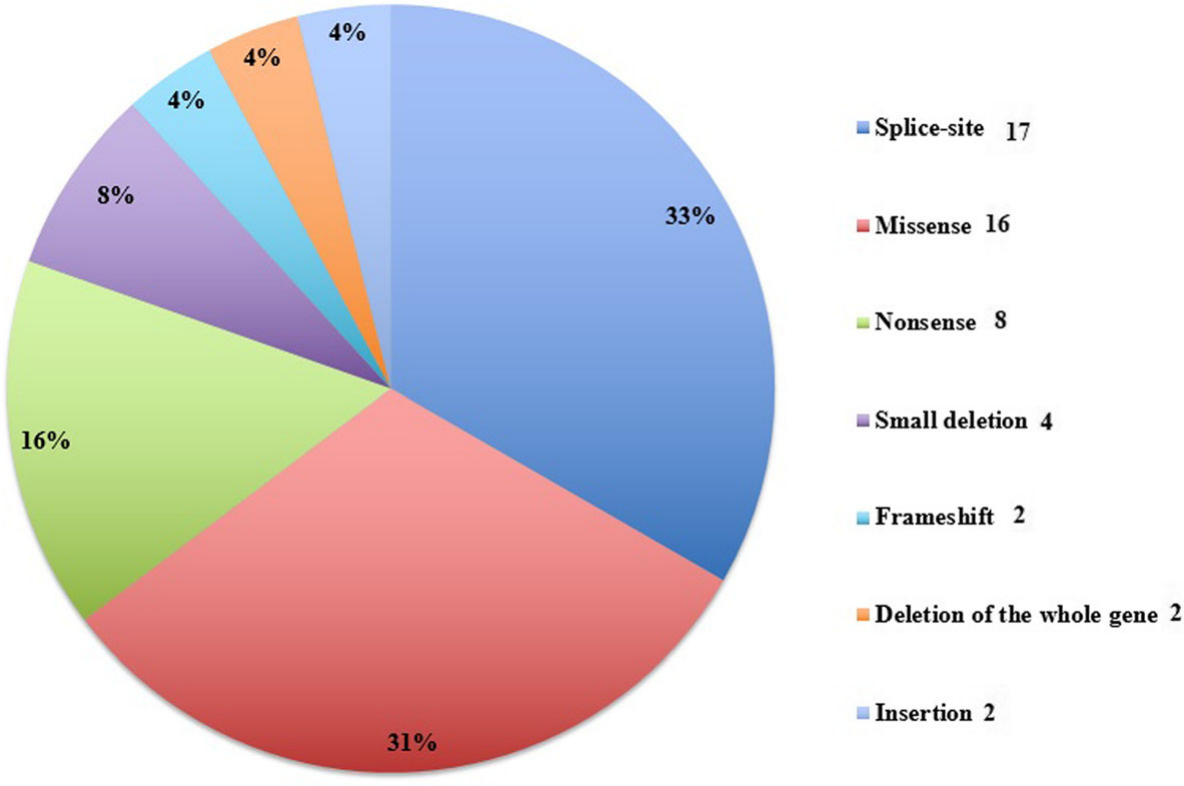
Mutations detected

Mutational analysis has been performed in 67 patients, and resulted positive in 51 (76%). The mutational screening of the *NF1* gene revealed different mutations including: 8 nonsense mutations (15.7%), 2 deletions of the whole gene (4%), 4 small deletions (7.8%), 2 frameshift mutations (4%), 16 missense mutations (31.3%), 17 splice-site mutations (33%) and 2 insertion mutations (4%) (Fig. 4).

Twenty-four of 51 mutations detected in our patients have not been described to date, and are shown in Table 3.

**Table 3:** New mutations described

New mutations described					
Missense	Nonsense	Splice-site	Frameshift	Small deletion	Insertion
c.1318 C > T	c.4648G > T (p.Glu1550X)	c.6757G > T	c.27_28delGG	c.1756_1759delACTA	c.6791-6792insA
c.6757G > T	c.5149A > T (p.Lys1717X)	c.6782C > T			
c.5992G > C		c.2709G > A			
c.5482G > T		c.1228_1260 + 3del36			
c.5546G > A		c.1527 + 1G > T			
c.3112A > G		c.3871-1G > T			
c.1316 T > C		c.288 + 5G > A			
c.4268A > T		c.3974 + 2_3974 + 3delTTinsGG			
c.2709G > A		c.1261-1G > C			
c.7694C > G					

## Discussion

Our analysis was preliminary directed towards the definition of the phenotype of a cohort of NF1 patients. Our patients age varied from few days to 60 years, with a mean age of 13 years. This low average age may have determined an underestimation of major clinical features, whose appearance is age-dependent [11]. According to previous reports [12], we found CALs in 92% of patients. CNFs/SCNFs and plexiform neurofibromas were also detected in a considerable number of subjects (about 30% considered together). Either for skin manifestations or neurofibromas no gender preference was noted.

Ocular involvement has been recorded in a smaller number of patients than reported in previous studies. Lisch nodules and optic nerve gliomas were present in 10% and 3.5% of subjects respectively.

Conversely, the characteristic NF1 bone lesions were identified in a higher rate of patients than reported in the literature [4]. Phenotypical spectrum of the skeletal lesions described in NF1 was widely represented (tibial/sphenoid dysplasia, lordo/kyphoscoliosis, osteofibromas, pseudarthrosis) in the present cohort, and enriched by further clinical features not commonly reported (valgus knee/ft, *pectus excavatum*) [12]. A clinical association has been found between patients carrying sphenoid dysplasia with those showing recurrent sinusitis, and neuroradiological findings of mucosal thickening of the sinuses [13]. We suggest that such facial skeletal alteration, involving the big and/or small wings of the bone with its subsequent distortion, may be the basis of local inflammation responsible for the clinical-radiological findings of sinusitis. The underlying pathogenic mechanisms of the skeletal defects are not fully understood and could have a multifactorial etiology. Recent studies showed that during ossification, one of the most important functions of neurofibromin is to regulate osteoprogenitors as well as composition of the bone matrix [5]. Additionally, haploinsufficiency of neurofibromin leads to premature apoptosis of osteoblasts and alteration in proliferation/differentiation of osteoprogenitor cells. Experimental evidence suggests also that osteoblasts are deficient and osteoclasts have increased survival rates, leading to enhanced degradation of bony tissue [6].

According to previous report [14] in our patients up to 4 years of age, developmental delay and or preschool learning difficulties were found in more than 50%, while in patients 5 to 10 years of age a cognitive impairment responsible for school difficulties was reported in more than 35%. We cannot rule out that subjects with normal cognitive level, may not manifest some cognitive impairment or learning disabilities in the following years.

A statistically significant male gender prevalence was found for developmental and or preschool learning difficulties in the age group up to 4 years, although previous reports showed a female prevalence [8]. This observation could be linked to other and unknown genomic and/or epigenetic factors responsible for neurodevelopmental disorders in NF1 patients.

Headache, seizures and hydrocephalus were recorded in few cases. Brain abnormalities related to NF1 were not present in the 4 epileptic patients (1 case of absence epilepsy, 3 cases of generalized tonic-clonic epilepsy). Hydrocephalus observed in 3 patients resulted from Sylvian aqueductal stenosis, a very rare and severe complication described in NF1 patients [7, 12].

Among the neuroradiologic findings revealed in the present series, UBOs and tumors (optic nerve gliomas and acoustic neurinomas) were the most frequent. Our data confirm previous reports showing very low prevalence of tumors other than brain gliomas [4, 8, 15]. Coversely, no correlations between the presence of a splice-site mutation and the occurrence of tumors was found.

Optic nerve gliomas represent the almost totality of tumors [12]. Although these lesions are prevalently benign, a careful clinical monitoring is necessary for the minimal but possible risk of progression.

All patients included in this study underwent cardiological evaluation. After echocardiographic screening, a mitral valve insufficiency was found in 1.7% of patients and interatrial defect/patent *forame ovale* in 4.4% of subjects over 1 year. However, these heart defects are also frequent in the general population, and their presence in our sample could be purely coincidental.

Angiomas are rarely observed in NF1. Although they were identified only in 1.7% of our patients, they could have a pathogenic link with NF1. Indeed, impaired *NF1* gene function in vascular endothelial cells results in increased proliferation and growth, and could contribute to understand the vascular dysplasias usually affecting CNS [12].

The age at diagnosis had a unimodal distribution curve. It progressively increases till the end of pediatric age, and then gradually decreases. More diagnosed (70%) age group was 5–18 years, and the less ones were 30–60 years and 0–1 year accounting for the further 7% and 5%, respectively.

Twenty-four of 51 mutations found in this study have not been described to date. Among them, 16 are missense mutations; 2 gross deletions involving also neighboring genes include *NF1*, while other 4 deletions are small mutations; 2 insertion, 2 frameshift, 17 splice-site and 8 nonsense mutations were also detected. All these mutations lead to abnormal neurofibromin production (truncated/shortened) with premature terminations. Therefore, it could be assumed that also the novel changes are likely to be pathogenic and may affect NF1 protein function, as already evidenced in genetic studies showing that more than 80% of mutations previously described cause truncation or shortening of the gene product [16–18]. Nonsense, splice-site and missense mutations were the most frequently observed ones (80%), while insertion, deletion and frameshift were the less frequent (20%).

To date, there has been little evidence suggesting genotype-phenotype correlation in NF1, except for NF1 patients with a *NF1* microdeletion in which more severe clinical phenotypes, including a higher prevalence of learning disabilities and dysmorphic features, were found [17, 19]. This is consistent with the present report of 2 patients bearing large deletions with severe clinical phenotypes (including dysmorphic features, neurologic and skeletal abnormalities). In addition, as might be expected from the high mutation rate observed for the human *NF1* gene, almost half of all identified mutations occur de novo [8, 20], as evidenced also in our cohort (in which the rate of sporadic cases is even higher). As well as such genetic heterogeneity, the marked phenotypic variation associated with NF1 even in individuals carrying the same *NF1* mutation (e.g. the 2 widely overlapping deletion patients of the present series), lead to suggest the presence of feature-specific modifier genes which are unlinked to the *NF1 locus* itself, epigenetic alterations and/or other environmental factors interacting with each other [8, 17].

## Conclusions

At present, diagnosis of NF1 is based on clinical assessment, when two or more of the seven NF1 NIH diagnostic criteria are present [14, 21, 22].

This study may contribute to give further insights into the clinical characterization of the disease. In addition, it expands the spectrum of mutations in the *NF1* gene.

Despite the development of several methods to screen *NF1* mutations [19], and the encouraging results from potential pharmacologic or biologic therapies in clinical trials [23], the current NF1 management is symptomatic, directed towards the treatment of various complications and includes genetic counseling [17]. Hence, identification and providing information of pathologic mutations are important, even more to sporadic patients who are usually diagnosed later than familial cases. Besides the direct-sequencing method of genomic DNA, supplementary tests (reverse transcription PCR of total RNA, MLPA, FISH), which have been used in the present study, can be helpful to identify specific genotypes of patients [20, 24, 25]. The ongoing recognition of different mutations may give insights into the still unknown mechanisms involved in the development of NF1, allowing further genotype-phenotype correlations.

Early progressive deformities, state of disability, learning difficulties, and cognitive impairment result in severe social handicap for these patients [17]. Therefore, they need an early multidisciplinary and individualized management.

## Abbreviations

a-CGH: Array comparative genomic hybridization
CALs: Café-au-lait spots
CNFs: Cutaneous neurofibromas
CNS: Central nervous system
FISH: Fluorescence in situ hybridization
MLPA: Multiplex ligation-dependent probe amplification
MR: Magnetic resonance
NF1: Neurofibromatosis type 1
NIH: National Institutes of Health
PCR: Polymerase chain reaction
SCNFs: Subcutaneous neurofibromas
UBOs: Unidentified bright objects
